# Bio‐Archive of Cultural Heritage Microbiomes for Sustainable Conservation in the Multi‐Omics Era

**DOI:** 10.1002/ggn2.202500046

**Published:** 2025-12-14

**Authors:** Xiaomei Fu, Fasi Wu, Xiaobo Liu

**Affiliations:** ^1^ School of Environmental and Biological Engineering Nanjing University of Science and Technology Nanjing Jiangsu China; ^2^ National Research Center for Conservation of Ancient Wall Paintings and Earthen Sites Department of Conservation Research Dunhuang Academy Dunhuang Gansu China

**Keywords:** bio‐archive, biodeterioration, cultural heritage conservation, microbiome, multi‐omics

## Abstract

Cultural heritage sites are commonly exposed to outdoor environments, resulting in severe damage to heritage objects from biotic and abiotic processes. Control of outdoor environments is impossible for heritage conservation, and we cannot prevent the abiotic processes. However, a variety of mitigation management can be developed for biotic damage, such as microbial colonization and biodeterioration. Over the past few decades, both conventional cultivation‐dependent and modern cultivation‐independent techniques have been employed to elucidate the microbiomes associated with the biodeterioration of cultural heritage. However, many studies are limited to segmentary analyses or simply stop at examining the community composition of the microbiomes, lacking solid evidence of microbial metabolism and biochemical reactions between microorganisms and heritage materials to support the core microbiomes associated with the biodeterioration. Here, we recommend thoroughly exploring the benefits of more advanced multi‐omics techniques for analyzing cultural heritage microbiomes. We propose establishing a professional open‐access database to standardize analytical procedures, integrating both culture‐dependent and culture‐independent approaches, and bio‐archive valuable information on the core microbiomes, including their biodeterioration mechanisms, timelines, causes, and environmental conditions. This bio‐archive of cultural heritage microbiomes will empower conservators and researchers worldwide to develop evidence‐based, sustainable approaches for cultural heritage conservation under environmental change.

## Graphical abstract

This review emphasizes the importance of a bio‐archive of the core microbiomes of cultural heritage as a reference for sustainable conservation, encompassing the scope of multi‐omics approaches and the identification of core microbiomes that integrate with environmental parameters. Beyond this, it suggests establishing a professional organization dedicated to managing and standardizing these valuable datasets, which are open access to global cultural heritage conservators, offering a forward‐looking perspective on multidisciplinary cooperation.

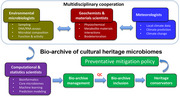

## 1 Introduction

**FIGURE 1:**
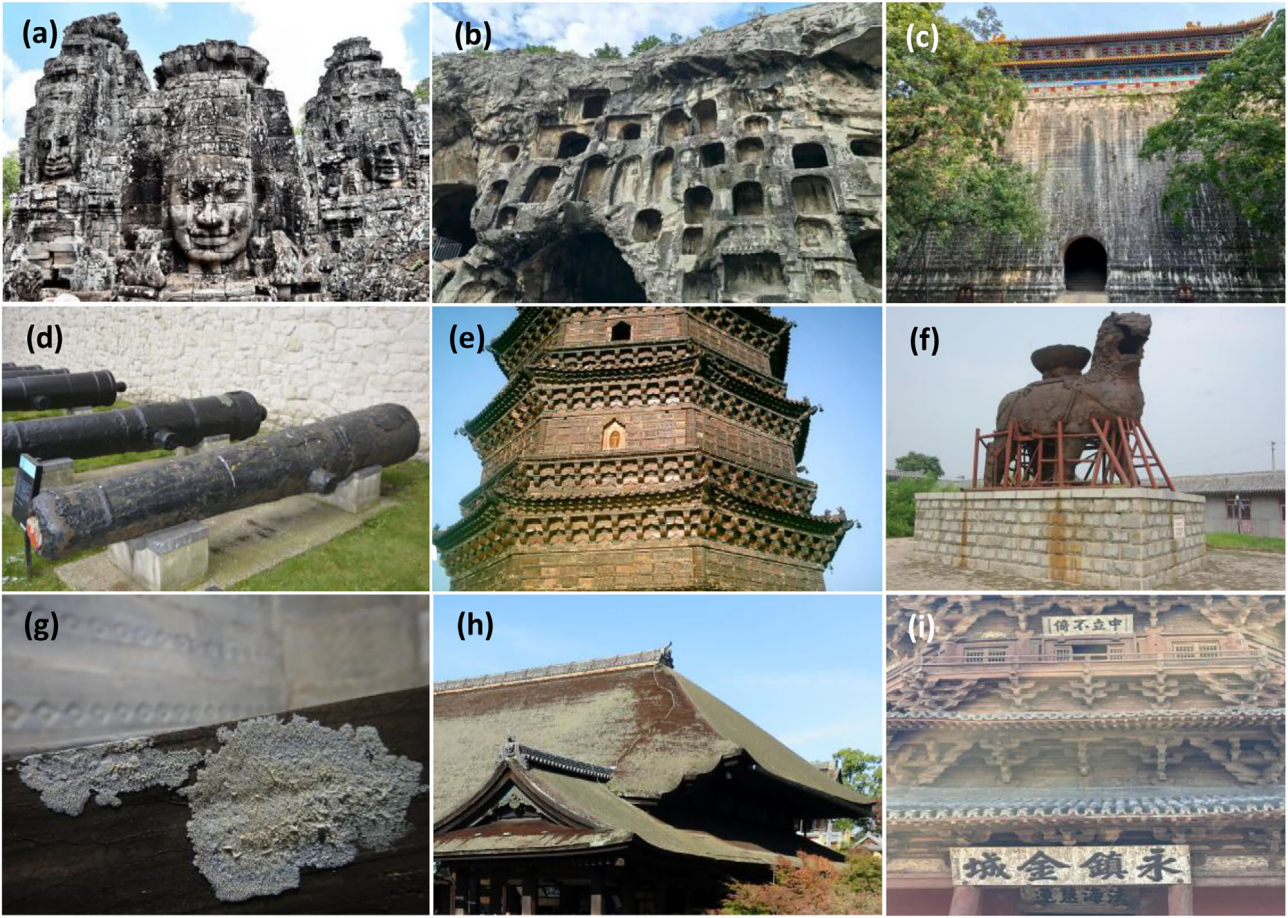
Examples of biodeterioration of outdoor cultural heritage materials of various types. (a) The four‐faced figures of the Bayon Temple at Angkor Thom, Cambodia. (b) Caves of the Longmen Grottoes, Luoyang, China. (c) Gate tower of Ming Xiaoling Mausoleum, Nanjing, China. (d) Guns at the White Tower, London, UK. (e) Iron Tower of Kaifeng, Kaifeng, China. (f) Iron Lion of Cangzhou, Cangzhou, China. (g) Da Gui Tomb of Luoyang, Luoyang, China. (h) Wooden roof of the Kiyomizu Temple, Japan. (i) Wooden Tower of Yingxian, Shuozhou, China. Images courtesy of F.W. and X.L.

Outdoor cultural heritage typically refers to immovable assets that encompass historical buildings, archaeological sites, grottoes, and stone monuments. Unfortunately, long‐term exposure to an open environment causes them to be displayed or stored in an uncontrollable condition that threatens their conservation and safety [1]. In this context, these objects are commonly affected by both abiotic and biotic issues worldwide (Figure 1). As abiotic loss or destruction is an inevitable process that involves the lifespan of heritage objects in nature, we cannot prevent or halt this process, especially in outdoor environments. However, conservators can try their best to mitigate biotic damage to cultural heritage, such as microbial biodeterioration, which is defined as any undesirable process caused by microbial activity [1]. Thus, it is necessary to devise a method to monitor the risks of microbial contamination that may occur on outdoor cultural heritage. The approaches applied to monitoring microbial risks include not only conventional technologies, such as visual inspection and microscopy, but also modern multi‐omics assays [2].

Prior to the development of high‐throughput sequencing technology, conventional techniques relied on the cultivation of microbial samples collected from archaeological sites. Previous studies that applied these cultivation‐dependent approaches have identified many isolates that may induce biodeterioration of cultural heritage objects [3, 4]. However, the majority of environmental microbiomes cannot be successfully cultivated using current technology [5]; therefore, it is impossible to determine the community structures and functions of these microbiomes accurately. Additionally, traditional molecular techniques, including DNA‐based fingerprinting, cloning, and Sanger sequencing, have been primarily applied to investigate and monitor microbial colonizers on cultural heritage objects in the past two decades [6, 7]. Although this molecular monitoring, combined with imaging and chemical analyses, has provided valuable insights into microbial risks to heritage objects of various materials, it yields limited data. It is time‐consuming and prone to bias. These shortcomings have hindered the advancement of monitoring microbial risks over the past few decades.

Fortunately, new high‐throughput molecular technologies help overcome such difficulties, enabling massive DNA sequencing and offering a much broader view of the real communities without the need for microbial cultivation and isolation. Among them, Next Generation Sequencing (NGS) technologies have developed through revolutionary improvements to address the complexities of environmental genomes and metagenomes, and are now well‐established in the study of cultural heritage microbiology [8]. These assays can be conducted using either the so‐called “shotgun metagenomic approach,” which sequences the entire DNA library of a given sample [9], or the “target amplification approach,” which targets specific, conserved sequences, such as 16S and 18S ribosomal RNA genes [10]. Notably, the latter approach enables semi‐quantitative analysis of specific functional genes or species, which is particularly useful in the field of cultural heritage microbiology, as DNA samples with poor quality or low concentration can be amplified using degenerate primers and PCR. Many of the studies using this strategy have been reviewed in recent literature [11].

Despite the advantages, the design of primers and/or exponential amplification introduces bias [12]. Furthermore, the amplification of ribosomal gene sequences does not provide any information on microbial functions. Nevertheless, third‐generation sequencing technologies are emerging and offering advantages over the limitations of NGS platforms [12]. A typical example is Nanopore sequencing technology, which utilizes protein nanopores embedded in a polymer membrane, eliminating the need for intervening PCR amplification or chemical labeling steps [13]. To date, nanopore sequencing technology has scarcely been applied in the field of cultural heritage due to its high testing costs. The recent study utilized Nanopore sequencing technology to perform a rapid diagnosis of microbial contamination, confirming the advantages that this new technology can offer for metagenomic analyses, particularly from very low DNA concentrations, and accurately reflecting the real proportions of all domains of life [14].

In recent years, the data generated by high‐throughput sequencing has increasingly expanded, informing sustainable mitigation measures to cope with microbial risks to cultural heritage objects. However, such information has not yet been collected and stored in a professional dataset, and the data analysis is not well standardized. Here, we emphasize the importance of a bio‐archive of the core microbiomes of cultural heritage as a reference for sustainable conservation of cultural heritage. The term “core microbiome” refers to any set of microbial taxa, or the genomic and functional attributes associated with those taxa, that are characteristic of the cultural heritage environment. A core microbiome is mainly determined by a combination of abundance‐driven metrics (that is, prioritize the most dominant members of the community, or those that have most effectively colonized cultural heritage) and occurrence‐based metrics (that is, require taxa to be present in every sample to be counted in the core) [15]. A number of ecologically and functionally important but low‐abundance taxa in the cultural heritage environment should also be considered. Moreover, identification of the core microbiomes requires uniform sequencing depth, standardizing the size of samples, and explicitly distinguishing spatial (that is, local, regional, and range‐wide cores) and temporal (that is, short‐term, seasonal, and multiyear cores) stability. Beyond this, we strongly suggest establishing a professional organization dedicated to managing and standardizing these valuable data, which are open access to global cultural heritage conservators.

## 2 Genomics‐Based Information on Cultural Heritage Microbiomes

**FIGURE 2:**
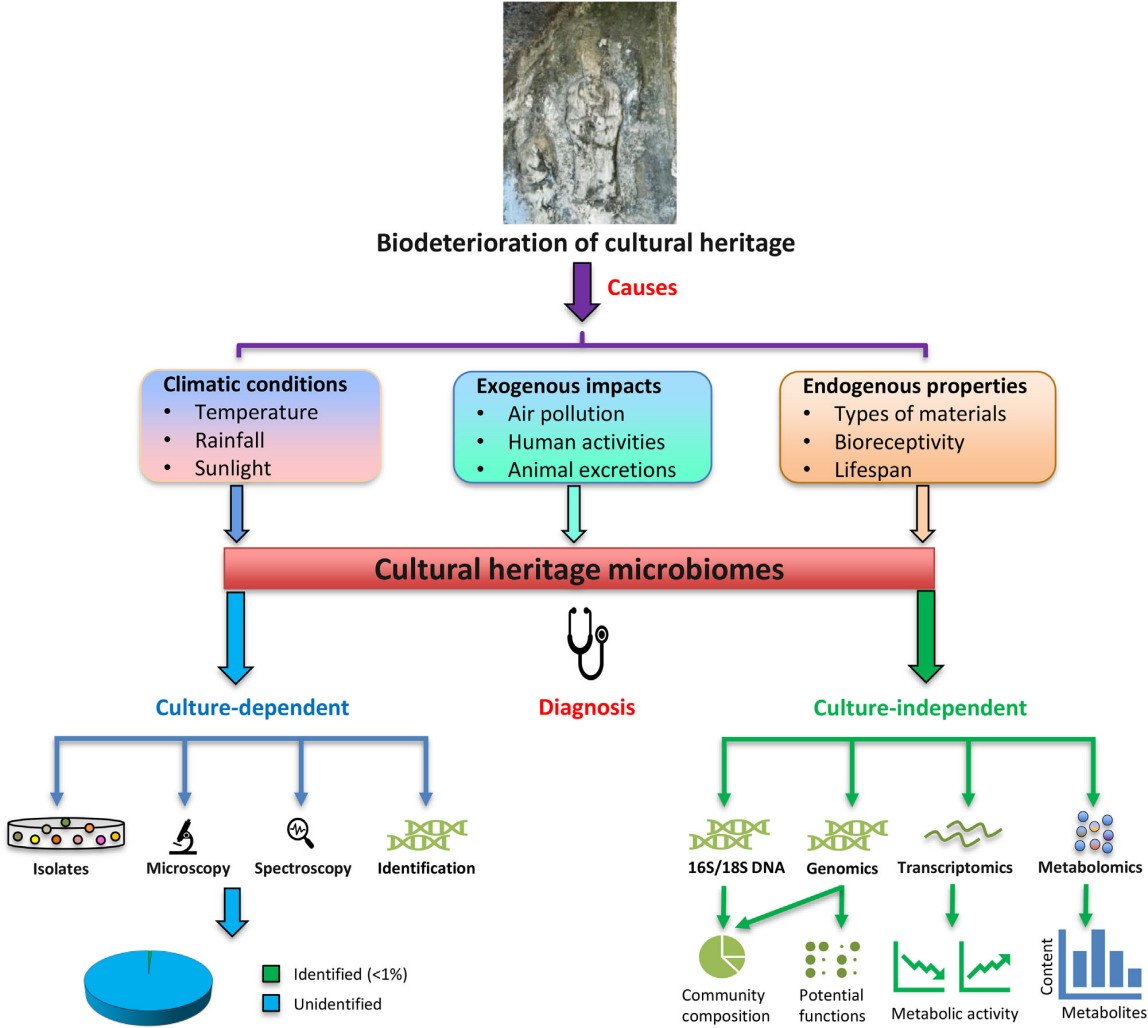
Schematic of cultural heritage microbiology investigation based on culture‐dependent and culture‐independent technologies. Microbial biodeterioration of stone heritage at the Longmen Grottoes archaeological site was used as an example.

Prior studies of cultural heritage microbiomes have commonly relied on culture‐dependent techniques, which detect and identify only culturable microbes by integrating molecular approaches and microscopy observations [16]. These studies have provided evidence that not only abundant signature isolates (e.g., bacteria and fungi) most likely derived from the surroundings, but also a variety of rare species whose origins were largely unclear. With the isolates, numerous laboratory‐based experiments can be conducted to confirm the biodeterioration roles of specific heritage materials [17], thereby helping to generate corresponding mitigation strategies to conserve the heritage. While these traditional studies made essential contributions to our understanding of cultural heritage microbiomes prior to the multi‐omics era, culture‐dependent approaches have limitations due to the typically small (0.1%–1%) proportion of microbiomes that can be identified [18], resulting in a significant amount of missing information on microbiomes (Figure 2). Moreover, potential biases are usually introduced due to the isolation of random species that are not members of the core microbiomes responsible for the biodeterioration of cultural heritage.

Over the past few years, we and others have introduced culture‐independent genome‐sequencing methods to unravel the community structures and potential functions of cultural heritage microbiomes [19, 20, 21]. These culture‐independent approaches provide valuable information on both culturable and unculturable microbiomes, allowing us to obtain a comprehensive profile of them rapidly (Figure 2). Notably, the accuracy of these approaches relies primarily on the setup and the amount of sampling collected. Comparison of different types of samples helps in understanding the core microbiomes that shape the divergence in biodeterioration processes taking place on cultural heritage. Thus, genome‐sequencing methods have been widely applied to cultural heritage microbiology in the era of multi‐omics.

There are numerous benefits to using genomics‐based approaches for diagnosing microbial diseases of cultural heritage, especially when rapid development of preventive conservation strategies is required under climate change. For example, we typically conduct systematic investigations into the seasonal successions of microbial communities and their functions using genomics‐based approaches [22, 23]. Metagenomic sequencing offers insight into the community composition and potential metabolic functions of all microbiomes, including Archaea, Bacteria, and Eukaryotes, enabling us to track temporal and spatial variations in cultural heritage microbiomes [21]. Having an overview of the predominant communities and potential metabolic reactions, we can correlate them with the physicochemical parameters of bio‐deteriorated heritage materials, thereby increasing the accuracy of the initial diagnosis of the core microbiomes responsible for biodeterioration. For example, we typically correlate the functional microbes that produce biogenic acids with dissolved ions (e.g., Ca^2+^, Mg^2+^, NO_3_^−^, and SO_4_^2−^) in bio‐deteriorated stone monuments to unveil the potential core microbiomes [24]. This feature, in conjunction with the large number of in situ physicochemical data obtained from repeated sampling and analysis, enables more detailed characterization and statistical analysis of the biodeterioration processes, which can provide insight into the annual variation of the core microbiomes [25]. Bio‐archiving of such information is crucial for generating effective preventive mitigation policies for seasonal outbreaks of microbial diseases. In short, genomics‐based assays are a valuable tool for directly observing the complete spectrum of community structure and functions that lead to the biodeterioration of heritage materials, as well as for guiding decision‐making on preventive measures.

Despite these benefits, genomics‐based assays cannot distinguish between inactive and active members in the microbiomes or confirm whether predictive metabolic functions are really occurring. Thus, we cannot fully determine the core microbiomes responsible for the biodeterioration of cultural heritage solely using genomics‐based information. In contrast, transcriptomics and/or metabolomics are particularly useful for discerning active communities and metabolisms in the microbiomes. This assay is a crucial step in identifying the core microbiomes associated with the biodeterioration of cultural heritage.

## 3 Transcriptomics and Metabolomics‐Based Evidence are Essential to the Core Microbiomes

Cultural heritage microbiomes are formed by both constituent organisms and their interspecific interactions. Microbial community transcriptomics enables the analysis of all transcripts produced in a microbiome. It is often used to investigate interactions between community members. This provides a better understanding of how microorganisms function together and the niches particular species may occupy. As a cultural heritage microbiome is a complex environmental microbiome, community transcriptomics is required to elucidate the total activity of the microbiome and identify active species that function in the biodeterioration processes [26].

Integrating with metagenomics assays, community transcriptomics provides information on the activity of the microbiome, explores the predominance of metabolic processes, and further confirms the potential core microbiomes associated with the biodeterioration processes [2]. For example, community transcriptomics focused on the functional genes responsible for biogeochemical cycles of carbon, nitrogen, and sulfur could greatly help understand the core microbiomes driving the biodeterioration of stone heritage [27]. Community transcriptomics of functional genes coding for enzymes involved in cellulose depolymerization or hydrolysis will help elucidate the biodegradation of cellulose‐based heritage materials, such as wooden objects, textiles, and written documents [28, 29].

**FIGURE 3:**
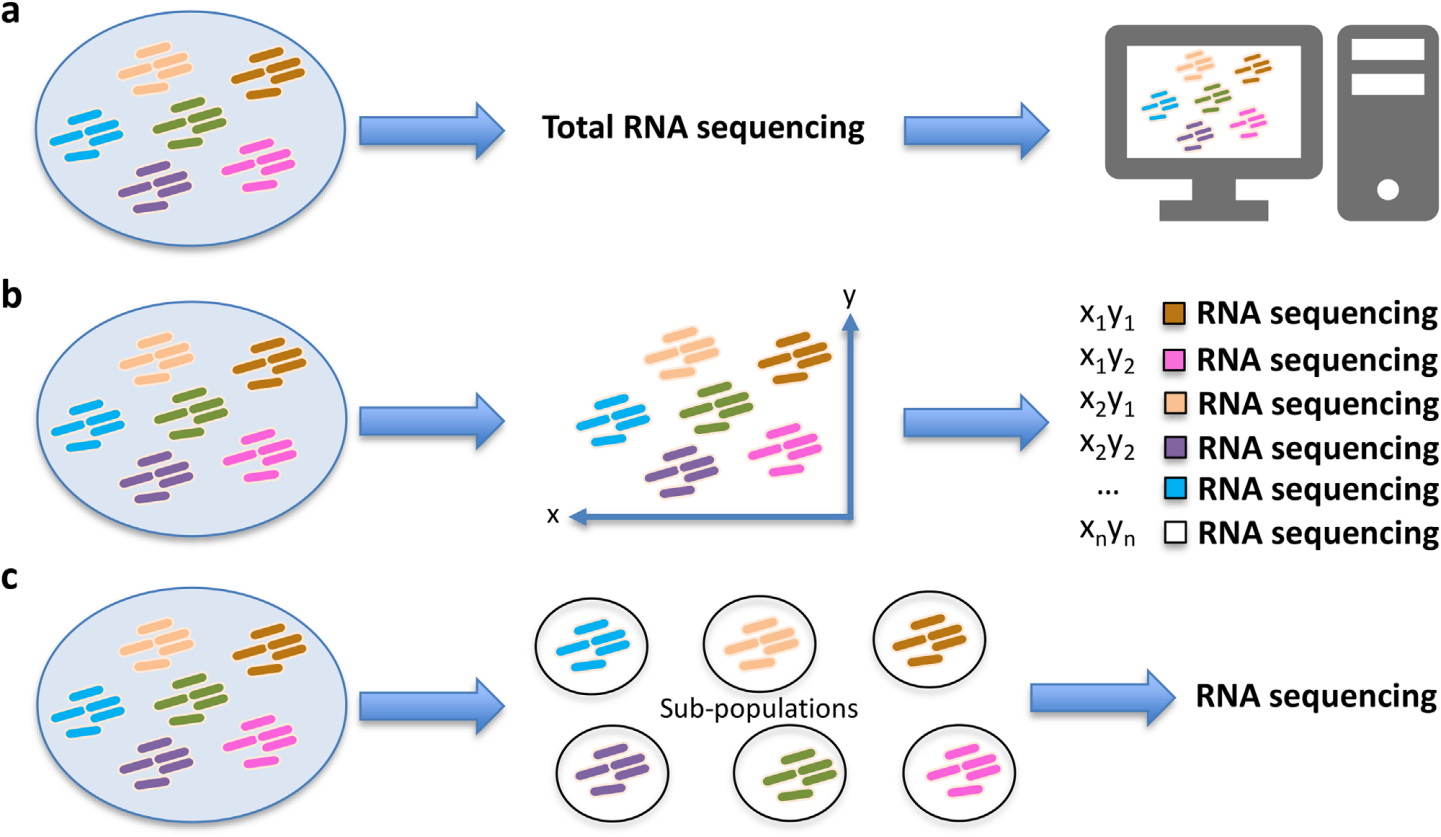
An overview of different community transcriptomics methods that could be used for cultural heritage microbiomes. (a) Meta‐transcriptomics: a method for capturing the transcriptomes of all the members of a microbiome at once. (b) Spatial transcriptomics: a method that combines high‐resolution imaging with transcriptomics analysis to measure spatial gene expression patterns within a bio‐sample. (c) Sorted transcriptomics: a method that first sorts the microbiomes into simpler sub‐populations to reduce the complexity of environmental microbiomes and then conducts transcriptional analysis.

However, environmental microbiomes are challenging to study, as they often necessitate de novo transcriptome assembly due to the typically unknown community composition and the potential predominance of poorly studied species [26]. The diversity of species can make it difficult, or even impossible, to culture samples. Although transcriptomics provides an overview of the functional expression of microbiomes, diverse community composition may also pose a problem for the accurate prediction of in situ cooperation across members and their specific contributions to biodeterioration. Furthermore, the presence of many unknown species in environmental samples complicates the steps of transcriptome assembly, species identification, and functional annotation, as many algorithms used in these steps require reference genomes. Despite these difficulties, several methods of transcriptomics have been applied to environmental community samples, such as meta‐transcriptomics [30], spatial transcriptomics [31], and sorted transcriptomics [32] (Figure 3). These methods could be applied to the transcriptomics of cultural heritage microbiomes if the sampling or total biomass in a sample is not a significant issue. For example, meta‐transcriptomics could be used to determine the entire activity of the microbiomes in bio‐deteriorated heritage materials, discern active communities and metabolisms, and unravel the core microbiome responsible for biodeterioration. Spatial transcriptomics is a valuable tool for elucidating the metabolic interactions between the layers of microbial communities within epilithic biofilms that colonize stone monuments. Sorted transcriptomics may be helpful in studying the organic carbon synthesis by phototrophs, such as cyanobacteria and microalgae, which are considered pioneers among colonizers on stone heritage.

Although community transcriptomics provides general information on ongoing metabolic reactions, the origin of chemical metabolites that are harmful to heritage materials is the direct evidence to confirm the core functional microbiomes that contribute to the biodeterioration of cultural heritage. Mass spectrometry‐based metabolomics is a key technology for detecting and identifying metabolites produced by the microbiomes, as well as understanding the functional roles of these microbial metabolites [33]. However, data analysis of chemicals is still an obstacle; therefore, the emphasis is placed on data analysis approaches and integrative analysis, including the integration of microbiome sequencing data [34]. Based on both metagenomics and transcriptomics‐based information, a metabolomics assay could focus primarily on the predominant metabolic processes closely associated with the physicochemical parameters of the samples, ultimately confirming the core microbiome that produces chemicals leading to biodeterioration.

Despite the necessity of transcriptomics and metabolomics‐based evidence to confirm the core microbiome, few studies have introduced these technologies to the analysis of the cultural heritage microbiome. One of the main issues is the restriction on the amount of biomass that can be collected from cultural heritage samples. Another issue is the complexity of microbiomes and metabolisms, which leads to difficulty in data analysis and often leaves much of the data unannotated due to a lack of a comprehensive reference database. However, this is a common challenge for transcriptomics and metabolomics when applied to environmental microbiomes. Multidisciplinary and integrative studies are therefore necessary to develop new bioinformatics methods that will enhance the comprehensive applications of these two techniques in cultural heritage microbiology.

## 4 Bio‐Archives of the Core Microbiomes Must Correlate with Environmental Parameters

Outdoor environments cannot be controlled, and microbial colonization and biodeterioration are markedly influenced by climate and location [35]. In the context of outdoor cultural heritage, exposure to an open environment can lead to the core microbiomes evolving in response to environmental changes [36, 37, 38, 39], such as atmospheric pollution, temperature, water, and sunlight. Thus, integrating with environmental factors is essential to the bio‐archives of the core microbiomes.

There is a particular need to assess and understand the impact of climate change on microbial colonization and biodeterioration of cultural heritage. Long‐term monitoring and recording of local climatic factors, including temperature, rainfall, and sunlight, can enhance the accuracy of bio‐archives of the core microbiomes under what climatic conditions they might bloom [40]. For example, there are clear demarcations in microbial colonization and growth of outdoor cultural heritage between different climatic zones, ranging from damp‐hot to arid‐cold areas [40, 41, 42]. Thus, it is essential to archive the core microbiomes according to the local climatic conditions. Notably, atmospheric pollution [43], domestic and industrial activities [44], and animal excretions [45] can further enhance the deposition of nutrients on cultural heritage, thereby selectively enriching potential functional microbiomes. Our previous studies have shown that bat excretions enrich ammonia‐oxidizing microbiomes (including archaea and bacteria) on sandstone monuments at the Angkor Wat archaeological site [46, 47]. Additionally, sulfur‐oxidizing bacteria are also abundant on the stone dogs of Leizhou, where volcanic eruptions have frequently occurred in the past [48]. The integration of environmental parameters will significantly aid in understanding the ecological succession patterns of the core microbiomes and inform preventive mitigation measures to address microbial biodeterioration in the long term.

By now, there is no standardized bio‐archive procedure for cultural heritage microbiomes. While a variety of methods are available for large‐scale investigations of cultural heritage microbiology, the development of best practices, standardized procedures, and ideal taxonomic approaches remains an ongoing challenge to ensure data quality and interpretation. To this end, standardized sampling, sequencing protocols, and bioinformatics analyses are required to obtain representative data and avoid sample processing biases before archiving them as a reference dataset for heritage conservators. Taking the case of biocrust investigation at the Longmen Grottoes site [49], we collect biocrust samples via non‐destructive methods (e.g., adhesive tapes) after fully recording the in situ information as an element of bio‐archive, package the biological samples (at least 6) with ice bags, and immediately send them to the laboratory for physiochemical analyses and DNA and/or RNA extraction. For physicochemical and genetic analyses, methods on the sample processing, chemical testing and DNA and/or RNA sequencing, bioinformatics‐based annotation, and statistical modeling for the core microbiomes will be recorded as a report for bio‐archive inclusion.

## 5 Microbial Bio‐Archives Should be Public to Cultural Heritage Conservators

To achieve the sustainable conservation of UNESCO World Heritage, the data from bio‐archives of cultural heritage microbiomes should be made public, as it is the responsibility of all humans. Details on the core microbiomes, including timelines, climatic factors, and heritage materials, should be documented in the bio‐archives to help heritage conservators take corresponding measures to cope with potential microbial diseases [50]. Additionally, any case that demonstrates an effective practice for controlling the core microbiomes, such as cyanobacteria on stone heritage [51] and fungi on wooden heritage [52], should be specifically detailed in the bio‐archives. In this case, we could provide a comprehensive archive that includes the detailed patterns of the microbial disease's occurrence on cultural heritage and effective mitigation management strategies. An international professional organization or department, such as UNESCO, could be established to check and manage the data before they are archived. To guarantee a good quality of the data, data standards (e.g., the size and origin of sample, sampling methods, in situ images, heritage materials types, and climatic conditions) and metadata requirements (e.g., sequencing depth, bioinformatics‐based procedure and reference database for taxonomic or functional annotation) should be clearly defined for this bio‐archive.

This is a challenging mission of bio‐archives of cultural heritage microbiomes, considering the diversity of heritage materials, locations, and climatic conditions. Interoperability with existing repositories, such as the World Heritage List (https://whc.unesco.org/en/list/) and WorldClim (https://worldclim.org/), could help achieve this goal. For example, retrieving information on climatic conditions and heritage materials will improve the prediction accuracy of the occurrence of microbial risks associated with the core microbiomes in the context of global climate change. Cooperation between the government and research institutes or universities is the foundation of cultural heritage conservation. Universities and research institutes are the primary contributors to fundamental research on the bio‐archives of core microbiomes and the development of feasible mitigation measures. The government is responsible for providing funds to support basic research and promoting effective practices that protect against microbial diseases. Based on this win‐win system, a good demonstration of cultural heritage conservation should be archived and promoted as a public reference worldwide. Only in this way can our cultural heritage conservation science and technology continue to advance in the future.

## 6 Conclusions and Future Directions

In recent decades, the conservation of cultural heritage has garnered increasing research interest from various scientific disciplines. Recently, the integration of modern multi‐omics techniques with cultural heritage microbiology has enhanced the efficiency of diagnosing and treating microbial diseases at sites of cultural heritage. The study of cultural heritage microbiomes requires a combination of different techniques. Multi‐omics techniques, including genomics, transcriptomics, and metabolomics, offer a new frontier in conservation, enabling the better study of the ecology, taxonomy, and interactions of microbiomes colonizing cultural heritage relics. In this context, data on cultural heritage microbiomes have expanded significantly worldwide. Unfortunately, such research data are scattered, unstandardized, and not publicly available to cultural heritage conservators. Therefore, a public global database is urgently needed to archive the information on cultural heritage microbiomes, especially in the multi‐omics era.

One of the most revolutionary advances in cultural heritage microbiology in recent years has been the realization that high‐throughput sequencing techniques, including genomics and transcriptomics sequencing, allow the culture‐independent study of microbiomes colonizing heritage relics. Moving beyond documenting the DNA‐based information present in these diverse environments of outdoor cultural heritage, the study of cultural heritage microbiomes is now focusing on defining the mechanisms underlying the interactions between microorganisms and heritage materials as well as their environment [2, 53]. One of the primary objectives is to elucidate how the composition and functions of the core microbiomes influence the initiation and progression of significant cultural heritage diseases, such as the biodegradation of wooden heritage and the biodeterioration of stone heritage, to enhance their diagnosis and treatment. Progress in the field has been driven by multi‐omics technologies, combined with new bioinformatics tools and models to interpret the vast complexity of this fascinating research area. This enables a bio‐archive of novel mechanistic insights into the composition and functions of the core microbiomes, ultimately advancing heritage conversion science and technology.

**FIGURE 4:**
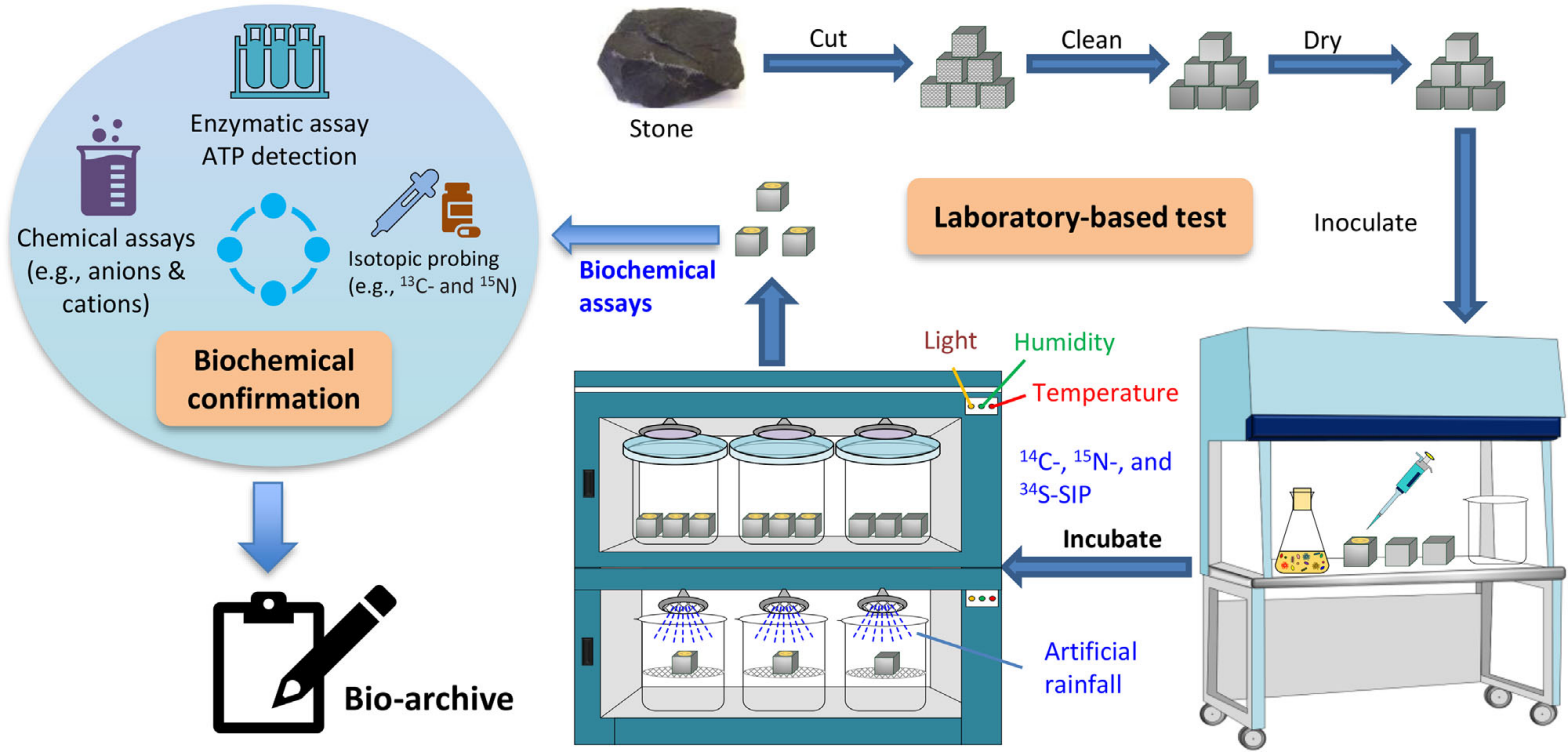
Laboratory‐based experiments for final confirmation of the core microbiomes before bio‐archiving. Microbial biodeterioration of stone heritage was used as an example.

**FIGURE 5:**
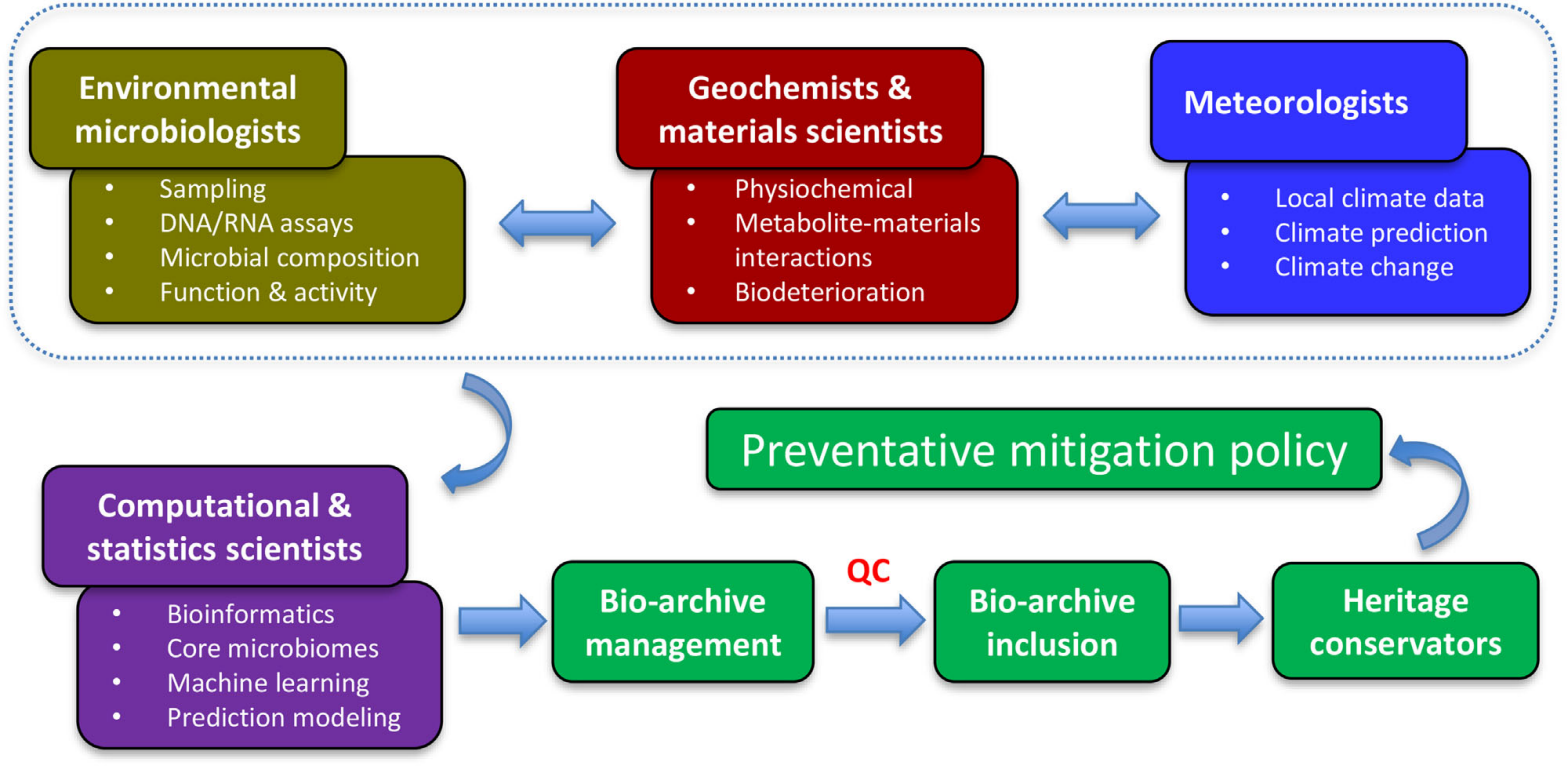
Schematic summarizing the proposed workflow and stakeholder roles of bio‐archive inclusion.

Although the multi‐omics revolution for cultural heritage microbiology is only just beginning, more exciting discoveries are expected in the future through the integration of genomics, transcriptomics, and metabolomics specialties with microbial cultivable techniques and restoration/conservation applications [11, 54]. Multidisciplinary approaches of environmental microbiology, geochemistry, materials science, meteorology and computational science are therefore key to this bright future and essential for unraveling the role that core microbiomes can play in cultural heritage conservation, from the viewpoints of both biodeterioration and bioprotection [36, 37, 55, 56]. For example, the endeavor in laboratory‐based experiments is to test the occurrence of real chemical reactions (Figure 4), including ongoing metabolic processes and interactions between metabolites and heritage materials. This will provide solid evidence to confirm the core microbiomes that are responsible for the biodeterioration of cultural heritage. Given this necessity, traditional culture‐dependent approaches should be integrated to test the series of chemical interactions, particularly the metabolite‐material interactions. Next, intensive collaborations of geochemists and materials scientists will generate the data about the core microbiomes responsible for specific biodeterioration processes. Upon the database in the bio‐archive, computational scientists and meteorologists using machine learning could be informed with effective models that accurately predict the occurrence of potential microbial risks under climate change. With such confirmed information, a bio‐archive of the core microbiomes responsible for the biodeterioration of cultural heritage is reliable for heritage conservators before making any preventative mitigation policy (Figure 5).

## Conflicts of Interest

The authors declare no conflicts of interest.

## Data Availability

The authors have nothing to report
